# Designing Data Elements and Minimum Data Set (MDS) for Creating the Registry of Patients with Gestational Diabetes Mellitus

**DOI:** 10.25122/jml-2019-0011

**Published:** 2019

**Authors:** Maryam Ahmadi, Esmat Mirbagheri

**Affiliations:** Department of Health Information Management, School of Management and Medical Information Sciences, University of Medical Sciences, Tehran, Iran

**Keywords:** Gestational diabetes mellitus (GDM), minimum data set (MDS), registry

## Abstract

The prevalence of gestational diabetes mellitus (GDM) is increasing in Iran. Collection of patients’ data is commonly conducted through using medical records. However, for providing a structured reporting based on the information needs, a minimum data set is a fast, inexpensive, and suitable method. For exchanging high-quality data between different healthcare centers and health monitoring organization, the data are required to be uniformly collected and registered. The present study aims at designing an MDS for creating the registry of GDM. The present study is an applied one, conducted in two stages, with a qualitative Delphi method in 2018. In the first stage of the study, it was attempted to extract the data elements of mothers with GDM, through reviewing the related studies and collecting these patients’ data from the medical records. Then, based on the results of the first stage, a questionnaire including demographic, clinical, and pharmaceutical data was distributed among 20 individuals including gynecologists, pharmacists, nurses, and midwives. The validity of the questionnaire was examined by a team of experts and its reliability was examined by using Cronbach’s alpha. Data analysis was conducted using descriptive statistics (frequency, percentage, and mean) and excel. An MDS of gestational diabetes mellitus was developed. This MDS divided into three categories: administrative, clinical, and pharmaceutical with 4, 18, and 2 sections and 35, 199, and 12 data elements, respectively. Determining the minimum data sets of GDM will be an effective step toward integrating and improving data management of patients with GDM. Moreover, it will be possible to store and retrieve the data related to these patients.

## Introduction

Gestational diabetes mellitus (GDM) is an increase in a person’s level of blood sugar. GDM is either initiated or diagnosed during pregnancy [1]. In both cases, the pre-gestational and GDM are followed by unwanted complications that can bring about short-term and long-term consequences for mothers and their infants [2]. This disease is an increasing health problem in all communities, and it is considered as one of the most prevalent complications of pregnancy. According to the estimates provided by the World Health Organization, the prevalence of GDM will have been 1.5 times more than that of 2000. The prevalence of GDM has been reported to be 1–14% in different parts of the world [3]. GDM is associated with numerous complications for mothers and infants during pregnancy and delivery; it is distinct from the other type of diabetes i.e. non-gestational diabetes. The mother’s unwanted complications include abortion, preterm birth, preterm delivery pain, preeclampsia, cesarean delivery, and increased length of hospitalization, up to seven days or more. The unwanted complications for the infants include congenital anomalies, high birth weight, birth injuries, neonatal jaundice, low Apgar score, need for neonatal resuscitation, hospitalization in critical care nursing, and increased hospitalization length up to 7–13 days. Mothers with type 1 diabetes are dealing with a higher cesarean rate, high blood pressure, and preterm birth. Mothers with type 2 diabetes will suffer from a higher rate of stillbirth [4]. Also, GDM and hypertensive disorders are recognized as risk factors for premature maternal cardiovascular disease (CVD) and associated with other pregnancy-related complications and outcomes, such as pregnancy losses [5].

The significance of data collection in GDM indicates the increasing awareness of short-term and long-term effects of this disease for both mothers and infants. A minimum data set for reporting diabetes during pregnancy will result in a better understanding and monitoring of risk factors and their results, including different types of GDM. Moreover, a minimum data set is likely to indicate the significance of monitoring health changes in the population, the effect of gestational interventions, and the services required by pregnant mothers [6]. Given the importance of the target population i.e. promoting the health quality of pregnant women and controlling and treating GDM is of high significance [7]. Over recent years, the researchers have taken into account the potential applications of medical records in clinical and patients’ immunity researches, including data collection for clinical trials, reports on unfortunate events, and epidemiological studies [8]. The content of the data collected including observations, interpretations, projects, measures, and results depend on the completeness and accuracy of the data [9]. Helping the decision-making process, saving time, and providing the cooperation of all components of care are the main results the achievement of which calls for data exchange among different systems [10]. The main advantages of applying a minimum data set are the formation of information policy, as well as monitoring the health systems and providing the possibility of further studies for researchers. These data are finally regulated and presented in the form of statistical information, reports, hospitalization trend analysis, daily health services on a national macro level based on the care providers, and financial goals [2].

Furthermore, in order to create a system that is completely compatible with data exchange, it is primarily required to agree on the data elements set [11]. The agreement for creating and applying a minimum data set will allow policymakers, planners, software experts, and health data managers to know what data should be collected in the information system while they start designing a system [12]. There are numerous problems in our country including the lack of attention to the registration of data related to pregnancy cares, the existence of duplicate data elements on health forms, lack of timely access to medical records, lack of integrated data systems in medical centers, and lack of standard minimum data sets [13]. For having electronic data and saving at databases, it is essential to use an MDS [14].

In their study titled “Results of the first recorded evaluation of a national GDM mellitus register: Challenges in screening, registration, and follow-up for diabetes risk”, Boyle et al. state that the registration of a large number of patients in the Australian GDM registry will result in the provision of sufficient and accurate data for finding the risk factors, having an early diagnosis, improving screening goals, improving treatment results, correcting the lifestyle, and reducing the risk of suffering from post-gestational type 2 diagnosis [15]. Creating a minimum data set for collecting integrated and standard data is the most important measure to be taken [16]. According to Common Clinical Data Set by The Office of the National Coordinator for Health Information Technology, demographic data, clinical data, procedures data, medications data, and identifier data related to patients should be gathered [17].

At present, there is no summary of the information related to patients with GDM in the form of minimum data set for Iran. For collecting high-quality data and creating a system of integrated data as registry, the integration of data is essential for monitoring the status of maternal and neonatal health for two main reasons. Creating a minimum data set for GDM can be regarded as the first step for creating a national registry system of such data. Thus, the present study aims at creating a minimum set of administrative, clinical, and pharmaceutical data of GDM. It is also attempted to apply the same data with purposes of research, education, and pregnant women’s health status monitoring, prevention, and control of this disease by using its statistical results.

## Materials and Methods

**Figure 1:**
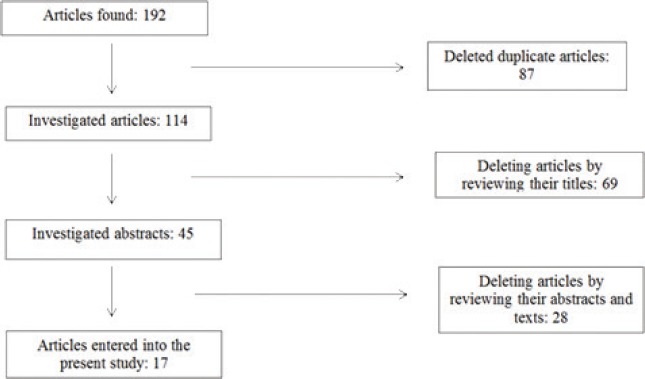
The process of selecting final articles from the investigated databases Literature Review

The present study is an applied one conducted in two stages with a qualitative Delphi method in 2018. In the first step, a review of the literature was conducted to retrieve related data resources. The resources included articles, reports, and forms available on the internet. In this step, a checklist was also used for the extraction of data elements. Searching the articles was conducted on Elsevier, Scopus, PubMed, SID, MagIran, ProQuest databases as well as Google Scholar search engine from 20072017. In the present study, all articles related to minimum data set, registry, and common data elements of GDM were examined and the main data elements were extracted. Sampling was not performed at this stage, and all the relevant literature was retrieved and evaluated based on the inclusion criteria, was then evaluated and the literature review was limited to the English language between 2007 and 2017, in full text from valid sources. The articles, whose full texts were impossible to access in addition to letters to editor, forms, and reports retrieved from websites, were excluded. Literature review was continued until data saturation. Then their desired data elements were entered into the checklist. Materials relevant to the subject were found using a search strategy (Figure 1).

Data was also collected from medical records of patients hospitalized due to GDM in ShahidAkhbarabadi Hospital, affiliated to Iran University of Medical Sciences and Arash Women’s Hospital, affiliated to Tehran University of Medical Sciences, two maternity hospitals in Tehran which having been ranked O14 in the ICD-10 classification. Medical records of these patients contained clinical, demographic, and discharge documents that were completed and archived. In order to validate the study, medical records of the patients of Mahdiyeh Educational Hospital affiliated with ShahidBeheshti University of Medical Sciences in the city of Tehran, which is a specialized hospital in obstetrics and gynecology were studied as well. In hospitals, 10 medical records in each O14 category related to GDM based on International Classification of Diseases 10th Revision (ICD-10) were randomly selected. In addition, data elements of emergency forms in hospitals were studied. In order to extract the data elements from the sources listed above, a checklist was used.

Collected data were divided into clinical, administrative, and pharmaceutical categories using a checklist. Then, extracted data elements from the literature review and patient medical records, in Iran, were combined and the final content of the checklist was constructed. The administrative data were classified into four categories. Moreover, the clinical and pharmaceutical data were classified in 19 and two categories, respectively. The questionnaire was constructed using the data elements of the mentioned checklist. The questionnaire was composed of five columns with, strongly agree, agree, neutral, disagree and strongly disagree in front of each data element. At the end of each section, a blank row was considered for adding necessary data elements by experts. Content validity of the questionnaire was evaluated by four experts, including two health information management experts, and two obstetrics and gynecology specialists. Test– retest reliability (with a 10-day interval) was performed to determine the reliability of the questionnaire. The collected data was analyzed using SPSS 19, and a Spearman’s rank correlation coefficient of 84% was achieved. To determine the final data elements of the MDS of the registry related to GDM, data elements were chosen by 20 samples of attending experts through the Decision Delphi technique in two rounds. The research environment was the workplace of gynecologists, pharmacists, nurses, and midwives working at two maternity hospitals in Tehran. These two hospitals were selected because they were teaching-medical hospitals and they accept a high number of pregnant women. The criteria for selecting the experts were being a faculty member and having at least five years’ experience in pregnancy diseases in clinical environments and hospitals. The second group was also required to consist of faculty members working in medical centers and having at least five years’ experience. The third and fourth groups were nurses and midwives working in maternity hospitals with at least five years’ experience. As many as 40 questionnaires were distributed among the experts of the four fields in two rounds (20 questionnaires in the first round and 20 questionnaires in the second round). All of the 40 questionnaires were completed and collected. Table 1 shows the attending experts demographic characteristics.

**Table 1:** Demographic characteristics of participants in decision Delphi technique

Participants	Numbers	Gender	Age group	Education	Academic field	Experience
**Gynecologist**	5	Female:5	20-29:2 30-39 :2 40-49 :1	Specialist:5	Gynecologists	5-10:3 > 10:2
**Pharmacist**	5	Female:5	30-39 :2 40-49 :3	Specialist:5	pharmacist	5-10:4 >10:1
**Nurse**	5	Female:5	20-29 :2 30-39 :3	Nurse:5	MSc:1 BSc:4	5-10:4 >10:1
**Midwife**	5	Female:5	20-29 :4 30-39 :1	Midwife:5	MSc:1 BSc:4	5-10:3 >10:2

The criteria for the acceptance of data elements in the final MDS, was the agreement level of experts. Data elements with agreement levels less than 50% were excluded at the first round, 50–75% agreement levels entered the second round, and agreement levels more than 75% were accepted in the first round of the Delphi technique. In the second round, an agreement level of 75% was considered on each data element. In the end, final data elements of the MDS were achieved in two rounds.

## Findings

**Figure 2:**
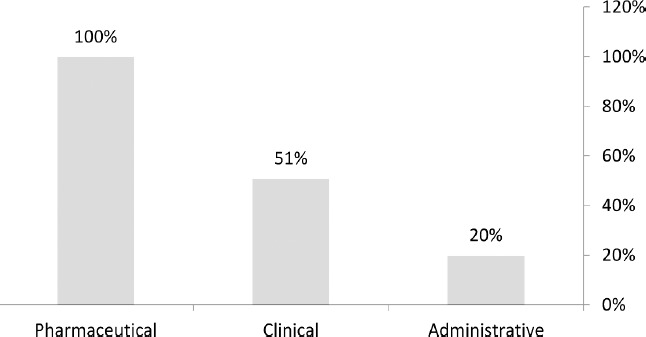
The percentage of the experts’ agreement in the first round of Delphi

The personal information of the experts, participating in the first and second rounds of Delphi, is presented in table 1. In the first round of Delphi, the experts agreed upon 20% of the administrative elements, 51% of the clinical elements, and 100% of the pharmaceutical elements (Figure 2).

**Figure 3:**
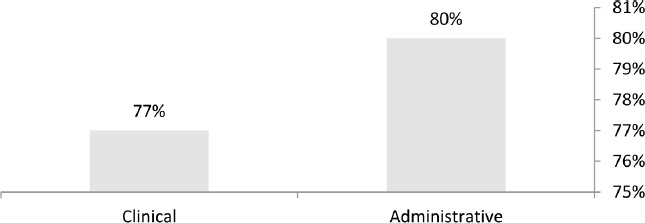
The percentage of the experts’ agreement in the second round of Delphi

However, in the second round of Delphi, the experts agreed upon 80% of the administrative elements and 77% of the clinical elements (Figures 3).

The suggested MDS of the gestational diabetes mellitus for Iran is indicated based on the section and the data elements of each category. The MDS of GDM was divided into three categories: administrative with four sections, clinical with 18 sections and pharmaceutical with two sections. The total number of final data elements for administrative, clinical and pharmaceutical categories was 43, 246 and12, respectively. After applying two rounds of the decision Delphi technique, the final data elements for administrative, clinical, and pharmaceutical categories were 35, 199 and12, respectively (Tables 2–4). 55 data elements, achieving less than 50 percent agreement, were removed from the MDS.

**Table 2:** Administrative data category for Minimum Data Set for GDM

Data sections	Number of data elements	First round of Delphi			Second round of Delphi			Final number of data elements
		<50%	50-75%	75%<	<50%	50-75%	75%<	
Demographic	18	0	15	3	5	0	10	13
Healthcare provider	14	0	11	3	2	0	9	11
Admission and its frequency	7	0	6	1	0	0	6	7
Patient’s indicators	4	0	3	1	0	0	3	4
**Total**	**43**	**0**	**35**	**8**	**7**	**0**	**28**	**35**

**Table 3:** Clinical data category for minimum data set for GDM

Data sections	Number of data elements	First round of Delphi			Second round of Delphi			Final number of data elements
		<50%	50-75%	75%<	<50%	50-75%	75%<	
Diagnostic data	6	0	0	6	0	0	0	6
Symptoms	9	0	0	9	0	0	0	9
Previous medical history & risk factors	20	0	12	8	9	0	3	11
Family history	4	0	4	0	4	0	0	0
History of previous deliveries	10	0	7	3	3	0	4	7
Physical and clinical examinations	17	0	3	14	2	0	1	15
Vaginal examinations	9	0	8	1	1	0	7	8
Disease prognosis in case of delivery	15	0	8	7	2	0	6	13
Laboratory data	31	0	24	7	9	0	15	22
Counselling	12	0	6	6	3	0	3	9
Permission for treatment and surgery	7	0	2	5	2	0	0	5
Current status of pregnancy	15	0	0	15	0	0	0	15
Pregnancy result	11	0	0	11	0	0	0	11
Prenatal and postpartum care for women with gestational diabetes	8	0	0	8	0	0	0	8
Anesthesia data	13	2	10	1	2	0	8	9
Sonography data	12	0	3	9	2	0	1	10
Blood sugar chart	5	0	1	4	1	0	0	4
Data related to the diabetic mother’s infant	29	0	22	7	6	0	16	23
Mother’s discharge data	13	0	6	7	0	0	6	13
**Total**	**246**	**2**	**116**	**128**	**46**	**0**	**70**	**199**

**Table 4:** Pharmaceutical data category for minimum data set of GDM

Data sections	Number of data elements	First round of Delphi			Second round of Delphi			Final number of data elements
		<50%	50-75%	75%<	<50%	50-75%	75%<	
Used drugs and substance dependence	5	0	0	5	0	0	0	5
Drugs used related to diabetes	7	0	0	7	0	0	0	7
**Total**	**12**	**0**	**0**	**12**	**0**	**0**	**0**	**12**

In the end, the administrative data elements were categorized in four sections- demographic, healthcare provider, admission and its frequency, and patient indicators. From 43 data elements, the experts agreed on as many as 35 data elements. (Table 5)

**Table 5:** Examples of administrative data elements for a minimum data set of GDM

Section	Data elements
Demographic	Patient name Patient family Nationality Date of birth (age)
Healthcare provider	Date of admission Hospitalization date Number of hospitalizations Hospital transfer Kind of admission
Admission and its frequency	Name of the hospital/healthcare center Hospital/health care center ID Address of the hospital/healthcare center Cause of reference Insurance data Insurance expiry date Patient’s costs Insurance costs
Patient’s indicators	The patient’s exclusive indicator Medical records number Admission code National ID number

The clinical data elements were categorized into 19 sections, viz., diagnostic data, symptoms, previous medical history and risk factors, family history, history of previous deliveries, physical and clinical examinations, vaginal examinations, disease prognosis in case of delivery, laboratory data, counseling, permission for treatment and surgery, the current status of pregnancy, pregnancy result, prenatal and postpartum care for women with GDM, anesthesia data, sonography data, blood sugar chart, data related to the diabetic mother’s infant, and the mother’s discharge data.

Finally, the experts agreed upon as many as 18 sections including 199 data elements. Given the experts’ lack of agreement, family history section and its data elements were removed (Table 6).

**Table 6:** Examples of clinical data elements for a minimum data set of GDM

Section	Data elements
Diagnostic data	Main complaint Primary diagnosis Diagnosis during treatment Final diagnosis
Symptoms	Pain/contraction Bleeding Leakage of fluid/rupture of membranes Fever Headache Edema of different organs Nausea
Previous medical history and risk factors	Cardiac diseases High blood pressure Kidney diseases Cervical cerclage
History of previous deliveries	Postpartum bleeding Delivery with forceps or vacuum extraction Abnormal fetus Post-term delivery Infants with a weight of more than 4000 grams
Physical and clinical examinations	Blood pressure Pulse Body temperature Breathing Starting time of pain Fetal heartbeat
Vaginal examinations	Dilatation Effacement Fetal position Fetal presentation Membranes
Disease prognosis in case of delivery	Kind of delivery Maternal general health Fetal general health Postpartum urinary status
Laboratory data	F.B.S. Glucose 2hpp BS(Stat) HbA1c FPG
Counselling	Date of request Kind of counselling Requesting doctor
Permission for treatment and surgery	Amputation permission Disclosure of information in legal cases
Current status of pregnancy	Number of pregnancies Number of abortions, Gestational age Probable delivery date Previous delivery method
Pregnancy result	Pregnancy termination Fetal death in week 13 Fetal death in week 13-23 Stillbirth (in week 24 and higher)
Prenatal and postpartum care for women with	HbA1c in the first trimester of pregnancy
Anesthesia data	Date of anesthesia Kind of anesthesia Kind of anesthetic Anesthesia duration Pre-anesthetic drugs
Sonography data	NST Amniotic fluid index Placental location Biophysical profile
Blood sugar chart	Post-breakfast PP2 Post- lunch HPP2 Post-dinner HPP2
Data related to the diabetic mother’s infant	Date of birth Time of birth Gender Birth weight (gram)
Mother’s discharge data	Primary diagnosis Diagnosis during treatment Final diagnosis Surgical operations

The pharmaceutical data elements were categorized into two sections, i.e., used drugs and substance use dependence, and drugs used related to diabetes. The experts agreed upon as many as 12 data elements, and they were used in the final MDS. (Table 7)

**Table 7:** Examples of pharmaceutical data elements for a minimum data set of GDM

Section	Data elements
Used drugs and substance dependence	Drugs that are currently being use Drug allergy Drug dependence Dependence on narcotics and type
Drugs used related to diabetes	The starting time of diabetes treatment diet therapy Oral Medication IV Medication IM Medication External Medication Internal Therapy Blood Products

## Discussion

The first stage for creating the registry of diabetic pregnant women, requiring constant follow-up, is identifying the information needs in medical centers. Based on the findings of the researcher, the data elements in paper records are commonly incomplete. Given the importance of such data, there are numerous shortcomings for having electronic data. After the poll, the table of the minimum data set was created for designing the registry of GDM. In these tables, the patients’ data were classified into three categories, i.e. administrative, clinical, and pharmaceutical (Table 2).

For determining the patient’s minimum administrative, clinical, and pharmaceutical data, in addition to searching the valid databases, the hospitalized patients’ medical records were examined as well. Moreover, by using the views of all individuals involved in the data collection of the present study (i.e., doctors, pharmacists, nurses, and midwives), the minimum data set required for creating the GDM registry was created. In this poll, as far as the administrative data section goes, the most important elements were the first name, last name, father’s name, marital status, date of admission, hospitalization date, number of hospitalizations, the name and address of the hospital/healthcare center, hospital/health care center ID, cause of reference, number of references, medical records number

As for the clinical data, the team participating in the poll determined the essential data. The data elements include symptoms of pain and bleeding, high blood pressure, fever, headache, the starting time of the pain, fetal approximate weight, maternal weight, BMI, and registered HbA1c in the first trimester of pregnancy, delivery lesions, Apgar score, intrauterine death cause (in case of occurrence), and other important elements.

In addition to the required data elements, given the experts’ views, the pharmaceutical set needed a separate classification for the essential data of pregnant mothers’ treatment duration. The most important data elements included the data related to current drugs, drug allergy, smoking dependence, smoking addiction, narcotics and their different kinds, the starting time of the diabetic treatment, pharmaceutical treatment/name/dose/prescription time: oral medication, IV/IM medication, external medication, internal therapy, and blood products. The experts agreed upon all of the aforementioned data elements.

In Australia, the Australian Institute of Health and Welfare has initiated the project of “The Pregnant Women’s Dataset” in the form of a minimum data set. One of the most important parts of the present project is collecting minimum data acquired from screening and caring for pregnant mothers with GDM [18]. In this data bank, the data are collected regarding the type of diabetes (type 1, type 2, and gestational), kind of pharmaceutical treatment, lifestyle, diet, sports, and lifestyle management. The data related to some of the aforementioned elements are the same as those of the present study.

Although the Australian Perinatal National Minimum Data Set contains information about gestational diabetes mellitus, these data have been conducted by applying different methods in different fields. This makes it difficult to compare them on a national level. Thus, applying standard definitions or similar classifications through a minimum data set of GDM is the first step in improving the adaptability of the reports and comparing the data on a national level [19].

In a study titled *“*Diabetes in pregnancy outcomes: A systematic review and proposed codification of definitions”, Feig et al. (2015) created a standard definition source for future studies. In the present study, the experts agreed upon the standard maternal and fetal data by examining the studies conducted within 2000–2012 as well other resources including the World Health Organization and the statements released by the related scientific communities. For reports of future studies on GDM and collection of the related data, the present study is highly recommended [20].

Sadoughi et al. (2015) have conducted an applied study titled “Minimum data sets of perinatal period for Iran: A Delphi study”. In their study, the minimum data sets of the perinatal period were collected by investigating the minimum data sets of the selected countries, viz., Australia, Canada, New Zealand, the USA, England, and Iran through applying library resources. Then, the recommended minimum data set was validated by providing the experts with a questionnaire. The recommended minimum data set of the perinatal period for Iran was classified in 15 sections. According to the present study, weak documentation and lack of standard data elements are the main integration problems of information systems. Moreover, the present study proposed designing and implementation of perinatal minimum data set [13]. The findings of the present study indicated that creating a minimum data set for GDM collects the data related to monitoring blood sugar and the fetal/neonatal status and provides them for the planners and beneficiaries to use them in electronic health records’ design and in future studies.

In their study titled “Results of the first recorded evaluation of a national gestational diabetes mellitus register: Challenges in screening, registration, and follow-up for diabetes risk” Boyle et al. (2018) collected the data from three GDM data centers to guarantee the integration of the data; all of the data related to pre- and post-partum cares as well as fetal/neonatal status were recorded as registry [15]. As in the study conducted by Boyle et al., the data of the present study included data about the number of pregnancies, neonatal birth date, maternal age at childbirth, the status of previous pregnancies, blood sugar status, HbA1C, GDM diagnosis (in case of existence), and demographic data, such as ethnicity, residential address and so forth.

## Conclusion

Given the priority of the Ministry of Health and Medical Education for developing the project of disease registration system, the health outcomes in Iran, and the high prevalence and incidence rate of GDM and its complications for the mother and fetus/infant, designing and implementing the registry system of GDM in Iran is of high significance. The minimum data set of GDM will result in the collection of the national data about the incidence and prevalence of the disease, care strategies and techniques, and the treatment provided for GDM patients. The minimum data set works as a platform for collecting the key data of a disease [21]. By accessing high-quality health data and overcoming the variety of the existing data in healing environments [22], it will be easier to improve the caring services for patients with gestational diabetes mellitus.

The analysis of the findings of the present study indicated that determining the minimum data set as the first step of GDM registration system’s implementation is of high significance for exchanging integrated health data in the healthcare industry. In fact, applying the minimum data set as the basis and foundation of GDM health registration system will result in designing the web-based electronic records corresponding to the social and health conditions of Iran and having quick access to accurate and comprehensive data of GDM. By planning, evaluating, and monitoring the status of the patients and identifying the shortcomings of providing medical services and its outcomes, it will be easier to arrange policies and plans for the gestational diabetes mellitus and its sufferers on a national level. The practical application of this minimum data set through forms of the GDM registration system for documenting the cares is likely to result in determining the validity and reliability of the data elements, based on the needs of the Iranian healthcare system. If necessary, the minimum data set can be updated according to the new medical protocols for GDM and the needs of the gestational diabetes mellitus registration system’s beneficiaries.

## Acknowledgment

This study has been funded and supported by Iran University of Medical Sciences (IUMS); Grant No.96-04-136-31843.

## Conflict of Interest

The authors confirm that there are no conflicts of interest.
